# Salvianolic Acid B, a Potential Chemopreventive Agent, for Head and Neck Squamous Cell Cancer

**DOI:** 10.1155/2011/534548

**Published:** 2010-12-20

**Authors:** Yuan Zhao, Yinhan Guo, Xinbin Gu

**Affiliations:** ^1^College of Dentistry, Howard University, 600 W Street, NW, Washington, DC 20059, USA; ^2^Oriental TenGen Technology Development Co. Ltd., Department of D&R, P.O. Box 100078, Beijing 100078, China

## Abstract

Head and neck squamous cell cancer (HNSCC) is one of the top ten cancers in the United States. The survival rate of HNSCC has only marginally improved over the last two decades. In addition, African-American men bear a disproportionate burden of this preventable disease. Therefore, a critical challenge of preventive health approaches is warranted. Salvianolic acid B (Sal-B) isolated from Salvia miltiorrhiza Bge, which is a well-know Chinese medicines has been safely used to treat and prevent aging diseases for thousand of years. Recently, the anticancer properties of Sal-B have received more attention. Sal-B significantly inhibits or delays the growth of HNSCC in both cultured HNSCC cells and HNSCC xenograft animal models. The following anticancer mechanisms have been proposed: the inhibition of COX-2/PGE-2 pathway, the promotion of apoptosis, and the modulation of angiogenesis. In conclusion, Sal-B is a potential HNSCC chemopreventive agent working through antioxidation and anti-inflammation mechanisms.

## 1. HNSCC and Chemoprevention

**Figure 1:**
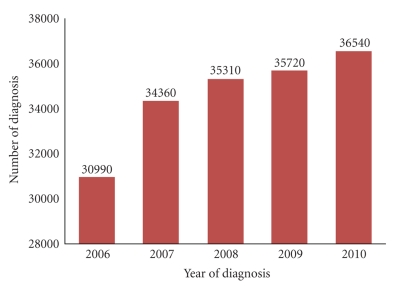
Incidence of oral cavity and pharynx cancer.

Over 90% of head and neck cancers are squamous cell carcinomas (HNSCC). Oral cancer accounts for a major proportion of HNSCC, which is the sixth most common cancer worldwide. In the United States, oral and pharyngeal cancers alone are diagnosed in about 36,540 Americans annually, and 7,880 are projected to die from these diseases in 2010 according to the American Cancer Society. HNSCC has been less studied compared to other cancers and the incidence of this cancer has not shown any improvement in the last 20 years (Figure 1). The 5-year survival rate for oral and pharyngeal cancers in Caucasian patients is 56%, while for African American men; it is only 34% [1]. In over 50% of first diagnosed cases of HNSCC in African American men, the cancer has already metastasized to other organs, such as the lungs. HNSCC prevention, earlier detection, and viable treatment options are of paramount importance to reduce the cancer incidence, improve patient outcomes and diminish the disparity. 

 HNSCC have been considered to be a typical multistep carcinogenesis with stepwise accumulations of genetic alterations resulting in aberrant cellular appearance, deregulated cell growth and carcinoma [2]. Patients with early stages of disease still have high risk to develop a second malignancy. A normal epithelial cell can take many years to undergo the multiple cellular and genetic alterations that lead to malignant changes. Thus, it remains an appealing strategy to develop effective, nontoxic and affordable novel pharmacological agents for preventing development of HNSCC and second primary HNSCC [2–5]. Chemoprevention has been considered a rational and appealing strategy to prevent or delay the development of HNSCC, additionally; dietary nutrients such as green tea, *β*-carotene and vitamin E have been also used as preventive agents [5–8]. Extensive studies have suggested that green tea, one of the most commonly consumed beverages worldwide, can reduce the risk of HNSCC development by inducing antioxidative activity via apoptosis and inhibiting epidermal growth factor receptor related signaling pathway [7, 9, 10]. There have been an increased number of case reports that high doses of green tea beverages cause hepatotoxicity [11]. Both vitamin E and *β*-carotene revealed the growth-inhibitory effect against lung cancer in cell culture and rodent models. But the promising activities have not translated into clinical success. Indeed, these supplements may actually lead to unexpected detrimental effects in humans as well as beneficial effects [12, 13]. Hence, the crux is to find an effective, nontoxic and affordable novel pharmacological agent in clinical trials for preventing carcinogenesis and the development of HNSCC as well as second primary HNSCC.

## 2. Salvianolic Acid B

**Figure 2:**
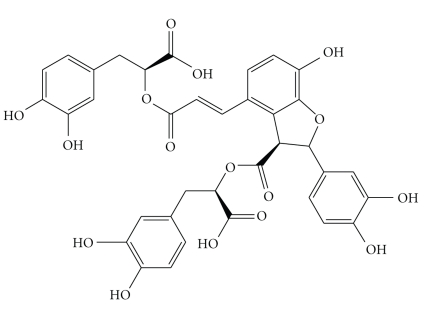
Chemical structure of Salvianolic acid B.

Radix Salviae miltiorrhizae (danshen or tanshen), the dried root of Salvia miltiorrhiza Bge is very important and popular in traditional Chinese medicine. It that has been widely and successfully used treating and preventing aging diseases, such as cardiovascular and cerebrovascular diseases, and cancers for thousand years and is ranked as a “Super grade” drug recorded in Shen-Nung's Pen-Ts'ao [14]. Currently, danshen has been accepted and used in Japan, the United States and some European countries [14–16]. In the last 50 years, Danshen received more attention by modern scientists that more than 70 compounds, including the hydrophilic depsides derivatives and the lipophilic diterpenoids, have been isolated from the Danshen herb [16, 17]. Salvianolic acid B (Sal-B) is the most abundant and bioactive member of the hydrophilic components in Danshen. Therefore, Sal-B is used as a quality control ingredient and active marker for S. miltiorrhiza Bge products by the National Pharmacopoeia Council of China. Sal-B contains seven phenolic hydroxyls which have been found to be closely related to redox potentials and/or antioxidant activities [18]. The structure of Sal-B is depicted in Figure 2. Sal-B has been studied for its preventive effects against cancer as well as cardiovascular, neurodegenerative, and other diseases [19–23]. The mechanisms mainly contribute to its antiinflammatory and antioxidative properties, modulation of apoptosis, inhibition of platelet aggregation, improved coronary microcirculation, as well as, regulation of angiogenic processes [14, 24, 25]. We will introduce the function and biological activities of Sal-B, validate its efficacy on HNSCC, and discuss the foreground of this component.

## 3. Antiinflammatory Activities

It appears that there is a general concept that chronic inflammation characterized by continued active inflammation responses and tissue destruction, can be a major cause of cancers and occur during the aging process [26–28]. Mounting studies have reported that Sal-B is capable of preventing the development of cancer; and the possible antiinflammatory mechanisms of Sal-B involve modulating cytokines, Cyclooxygenase-2/prostaglandin E2 (COX-2/PGE-2) pathway [24, 25, 29], NF-*κ*B [30–32], TNF-*α* [33–35] and MMPs [36–38]. Numbers of clinical experiences indicate its effectiveness and safety in contrast to the disadvantages of nonsteroidal antiinflammatory drugs (NSAIDs). NSAIDs, used primarily to treat inflammation, are associated with several serious side effects including gastrointestinal discomfort, cardiovascular disease and kidney failure [39–42].

### 3.1. COX-2/PGE-2

COX-2, an inducible form of cyclooxygenase is undetectable in most normal tissues, but abundant in the pathogenesis of inflammatory and neoplastic diseases. Additionally, its principal metabolite PGE2 has pleiotropic effects such as promoting cell proliferation, inhibiting cell death, promoting tumor angiogenesis, and decreasing immunosurveillance. Sal-B has been reported to attenuate significantly COX-2 expression and PGE2 production with or without lipopolysaccharide (LPS)-induced both in vitro and in vivo [24, 25, 29], and which may be attributed to the downregulation of JNK and ERK phosphorylation and blockage of MAPKs phosphorylation [29].

### 3.2. NF-*κ*B

NF-*κ*B, a protein complex that controls the transcription of DNA, regulates cellular responses as a “first responder” to harmful cellular stimuli, and its aberrant expression is linked to cancer and inflammation [43]. Several experiments clarified that the antiinflammatory effects of Sal-B depend on the inhibition of the NF-*κ*B signaling pathway [30–32]. Moreover, Sal-B attenuates the expression of VCAM-1 and ICAM-1 in TNF-*α* stimulated human aortic endothelial cell by partial blockage of NF-*κ*B expression [34].

### 3.3. TNF-*α*

Tumor necrosis factor-alpha (TNF-*α*), a representative proinflammatory cytokine damages cell structure and increases endothelial permeability and is involved in systemic inflammation [44]. Sal-B has showed to significantly reduced the production of TNF-*α* induced by LPS treatment in rat primary microglia in a dose-dependent manner [32]. In addition, Sal-B protects endothelial cell against TNF-*α* disruption by inhibiting vascular endothelial growth factor (VEGF) and extracellular signal-regulated kinase (ERK) activation [45].

### 3.4. MMPs

Matrix metalloproteinases (MMPs), inflammatory mediators are expressed in vascular cells in the course of atherosclerosis [46]. Sal-B treatment effectively inhibits MMP-2 and MMP-9 activitation and expression both cell culture and animal models, and it also, downregulates JNK and ERK phosphorylation [36, 37]. Some researchers found Sal-B could suppress high glucose-induced mesangial cells proliferation and extracellular matrix production in a dose-dependent manner, partially through modulating the cell-cycle progress and MMP-2 and MMP-9 activities [30, 31].

## 4. Antioxidative Activities

A vast amount of evidence suggests that overproduction of reactive oxygen species (ROS) and reactive nitrogen species (RNS) can damage cellular lipids, inhibit the normal function of proteins or DNA, and are associated with the pathogenesis of atherosclerosis, cardiovascular diseases, hypertension, ischemia/reperfusion injury, neurodegenerative diseases, and cancer [47, 48]. Sal-B, as an antioxidant neutralizes direct ROS attacks and terminates free radical-mediated oxidative reactions to protect the human body from such diseases [49]. 

### 4.1. Radical Scavenging

Reactive oxygen species (ROS), such as superoxide anion, hydroxyl radicals and hydrogen peroxide are chemically reactive molecules derived from oxygen. They usually contain one or more unpaired electrons in the atomic or molecular orbital [50]; therefore, they can easily participate in redox reactions and they play a crucial role in biological systems [51, 52]. Excess ROS can disturb the equilibrium status of prooxidant/antioxidant reactions, leading to the disruption of cellular functions in contrast to low/moderate concentrations that occur in response to induction of a mitogenic reaction and normal function of several cellular signaling pathways [53, 54]. There is increasing evidence that Sal-B has the capability to scavenging free radicals including superoxide anion, hydroxyl, DPPH (1,1-diphenyl-2-picryl-hydrazyl) and ABTS (2-azino-bis(3-ethylbenzthiazoline-6- sulfonic acid)) radicals in addition to hydrogen peroxide due to redox properties of the phenolic structure [55]. Sal-B showed a high antioxidant capacity in terms of neutralizing free radicals, as well as exhibiting significantly higher scavenging activity than l-Ascorbic acid (vitamin C) [56, 57]. 50% radical scavenging activity at a concentration of the Sal-B lower ~50% and ~40% than Vitamin C in DPPH and ABTS assays, respectively (1.81 ± 0.01 versus 3.44 ± 0.03 *μ*g/ml, 1.43 ± 0.01 versus 2.50 ± 0.02 *μ*g/ml) [57].

### 4.2. Antioxidant Activities

Free radicals have been elucidated to cause oxidative damage to cellular components, including attacks on DNA, oxidation of proteins and production of lipid peroxidation, these processes lead to disorder in cellular, tissue and organ function [52, 58, 59]. Sal-B has been reported to be a powerful and effective antioxidant, not only reducing lipid peroxidation, but also rescuing the loss of antioxidant enzyme activities against fibrosis and ischemia-reperfusion injuries [60, 61]. Some studies have reported that Sal-B can protect the brain and heart from ischemia-reperfusion injury by improving the recovery of motor function via regulating energy metabolism and maintaining the balance of free radicals such as SOD, GSH, and ATP levels against lipid peroxidation and superoxide anion production [62–65]. In the hepatic stellate cells (HSCs), Sal-B exerts suppressive effects on ROS to inhibit the proliferation and lipid peroxidation of HSCs through inhibiting NADPH oxidase and TGF-*β*1 secretion [49, 66, 67]. Studies have shown that ROS leads to the oxidation of low-density lipoproteins and accumulates within plaques and contributes to the atherosclerosis [66, 68]. Sal-B was identified to be a potent antioxidant, endothelial-protecting agent, an inhibitor that suppresses the expression of ICAM and VCAM, capable of inhibiting LDL oxidation and also inhibits ox-LDL induced endothelial injuries [69]. In sum, the antioxidative properties of Sal-B are closely associated with its protective effect of aging diseases [70].

## 5. HNSCC and Sal-B

Cancers have a close and delicate relationship with inflammation [26, 71, 72]. Inflammation often exists in the tumor microenvironment and is induced by inflammatory mediators produced by the tumor [73–75]. HNSCCs are highly inflammatory and aggressive, and they overexpress a number of inflammatory mediators such as COX-2, EGFR, VEGF and MMPs [76–78]. Sal-B, as chemopreventive agent exerts its effects by inhibiting tumor initiation and development; its anticarcinogenic activities have been clearly demonstrated in both cell cultures and animal models [24]. Furthermore, research has also shown that the combining Sal-B with other preventive agents is more effective than single-agent chemoprevention [25].

We tested the anticancer function of Sal-B in five human HNSCC cell lines (JHU-6, JHU-11, JHU-13, JHU-22 and JHU-29) that Sal-B significantly inhibit the cell growth in cultured cells [24]. In the animal experiments, HNSCC solid tumor volume in Sal-B treated group were significantly lower than those in untreated control groups [24]. The outcome is consistently obtained in human breast and prostate cancer cell lines. We found that Sal-B selectively suppresses COX-2-related mRNA and protein expression instead of housekeeping enzyme of COX-1 in the presence or absence of LPS stimulation. It seems that the chemopreventive effects of Sal-B depend on COX-2 expression levels, the higher the expression of COX-2 the more sensitive is Sal-B HNSCC cell growth-inhibition and PGE2 reduction. In addition, Sal-B induced caspase-dependent apoptosis by cleavage of a caspase substrate, poly (ADP) ribose polymerase (PARP). Sal-B also decreased the cellular amounts of antiapoptotic proteins such as NF-*κ*B, MDM-2, Bcl-2 and Bcl-xL and increased proapototic proteins such as p53 and caspase 3 [24, 79]. The mechanism of cancer-prevention was attributed to the COX-2/PGE2 inhibition and apoptotic pathway induction. PGE2, one of important prostaglandin product of COX-2 is involved in chronic inflammation [80].

A promising strategy to enhance the cancer-preventive efficacy is to use two anticancer agents in combination, which may produce synergy and lower the dose required for each agent [79, 81]. Celecoxib, a selective COX-2 inhibitor has been reported to have cancer-preventive effects in various types of cancers including HNSCC [82]. However, it was found to be associated with a dose-dependent cardiovascular morbidity that limited its long-term use. Hence, we decided to use Sal-B combined with low-dose celecoxib in order to increase the anticancer efficacy and reduce drug side effects. The outcome showed that the combination of half-dose of Sal-B and celecoxib greatly enhanced the inhibition of HNSCC cell proliferation compared with either Sal-B or celecoxib alone both in cell culture (JHU-06, JHU-011, JHU-013 and JHU-022) and tumor xenografts. The combination was associated with profound inhibition of the COX-2/PGE2/EGFR pathways, enhanced induction of apoptosis, and reduced the side effects of celecoxib due to dose reduction at the same time [25].

The anticancer effects of Sal-B were also found in 7,12-dimethylbenz-[a]anthracene-(DMBA) induced oral carcinogenesis in hamsters [83]. Experiments showed that Sal-B treatment significantly decreased the oral cancer incidence. Antiangiogenesis may be one of the possible mechanisms of inhibiting malignant transformation of oral precancerous [75, 84]. The formation of microvessels, and the expression of proangiogenic factors HIF1*α* and VEGF, was inhibited by Sal-B. 

**Figure 3:**
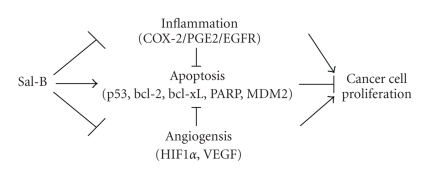
Possible anticancer activites of Salvianolic acid B.

Recently, we were inclined to accept the concept that the prevention is better than a cure. Actually, prevention is more valuable to reduce the incidence of HNSCC than increase survival rate [85, 86]. Anticancer properties of Sal-B are able to prevent and delay the malignant conversion of premalignant lesions and/or cell growth via inhibition of inflammation and angiogenesis and reduction of apoptosis (Figure 3).

## 6. Problem and Future Prospects

Sal-B as a popular compound of Traditional Chinese Medicine has been studied for its preventive effects against HNSCC. Most of the proposed beneficial effects have been attributed to antioxidative and antiinflammatory effects. It is known that the relationships among oxidation, inflammation and cancer are considered to be extremely complex. Firstly, chronic inflammation increase the risk of developing many types of cancer including HNSCC, and inflammatory cells, chemokines and cytokines are present in the microenvironment of all tumors [71]. Secondly, ROS, an endogenous class of carcinogens trigger the mutation of the cells have been demonstrated in the principal step of carcinogenesis and contribute to cancer progression. Moreover, cancer cells frequently produce more ROS than normal cells [87, 88]. Thirdly, activated inflammatory cells generate ROS and reactive RNS in response to proinflammaory stimuli, which can function as chemical effectors in inflammation-driven carcinogenesis; ROS-induced oxidations are implicated in inflammation via regulation signal pathways and related enzymes such as COX-2 [88, 89]. Sal-B, a natural anticarcinogenic agent with antiantioxidant and antiinflammatory activities, reacts easily with free radicals and inhibits effectively COX-2/PGE-2 pathway as well as regulating related cell signal pathways. It is difficult to distinguish which is determines prevention to HNSCC because oxidation, inflammation and cancer are intertwined in a complex web. Sal-B has been showed a significant advantage for the prevention and treatment in HNSCC due to effectiveness and nontoxic nature. 

The discovery of Sal-B anticancer properties is followed a path of from “bed” to “bench”. A thousand years clinical practices demonstrate that Danshen is able to effectively and safely prevent and treat aging diseases, such as cardiovascular diseases and cancers. The mechanism studies of Danshen have been concerned until latest 50 years. Sal-B is a most abundant and bioactive member of hydrophilic components in Danshen. The same as Danshen, Sal-B is a safer agent with no major side effects [20]. Due to our aging society, both cancer and cardiovascular diseases have increasingly become the two major killers and human health hazards. In recent years, the amount of cytotoxic agents and targeted therapies used to treat HNSCC, include classic chemotherapeutic agents, chemoprevention agents such as COX-2 inhibitors, monoclonal antibodies targeting tyrosine kinase receptors, small molecule tyrosine kinase inhibitors, and antiangiogenic drugs [90]. Unfortunately, these result in cardiovascular toxicity. Especially, celecoxib, a selective COX-2 inhibitor has been required restricted use owing to its potential to effects the cardiovascular system in long-term use by the Food and Drug Administration and was announced the early cessation of a cancer-prevention clinical trial. It is hailed that Sal-B is a not only cancer-preventive agent but an also cardiovascular protective agent. Sal-B inhibits cancer cell proliferation *in vitro* and *in vivo* in addition to regulating the microenvironment. In a study with celecoxib, Sal-B resulted in platelet aggregation, playing a key role in cardiovascular protection [20]. Since Sal-B exerts dual pharmacy characteristics, it will lead to broad applications in the future. But related-mechanisms are still not fully understood, we need specific and profound studies about Sal-B. 

In addition, some studies also reported Sal-B was released from nanotechnology samples faster and had increased antioxidant activity compared to the traditionally-powdered samples [89, 91, 92]. Combined with chemopreventive agents of traditional Chinese Medicine will further promote development of Sal-B.

We found an interesting and confusing phenomenon that Sal-B showed a distinct attack and protective features in different cell lines. Some studies reported Sal-B protected the SH-SY5Y neuroblastoma cells, hepatocyte and bone marrow stem cells against apoptosis by relieving oxidative stress and modulating the apoptotic process [93–95]; on the other hand, Sal-B has been revealed to activate apoptosis pathways in order to inhibit cancer cell proliferation [24, 25]. It seems to be controversial; whereas, the mechanism of signal regulation is very complex and therefore the role of Sal-B depends on the type of cell line, the microenvironment. Different concentrations of ROS showed both the induction and inhibition in cancer development. To define an accurate mechanism of Sal-B, particularly which are Sal-B exact targets, still calls for more studies and developments.

In conclusion, Sal-B, a natural antiinflammatory (selective COX-2 inhibitor) and antioxidative agent has chemopreventive activity on HNSCC; due to its effectiveness and safety it could have much more commercial value for food and medicine purposes.
